# Supplementary figures and images for: Financial Toxicity in Japanese Patients with Metastatic Renal Cell Carcinoma: A Cross-Sectional Study
Source: Cancers (Basel). 2024 May 16;16(10):1904. doi: 10.3390/cancers16101904 (PMC11119599; doi:10.3390/cancers16101904)

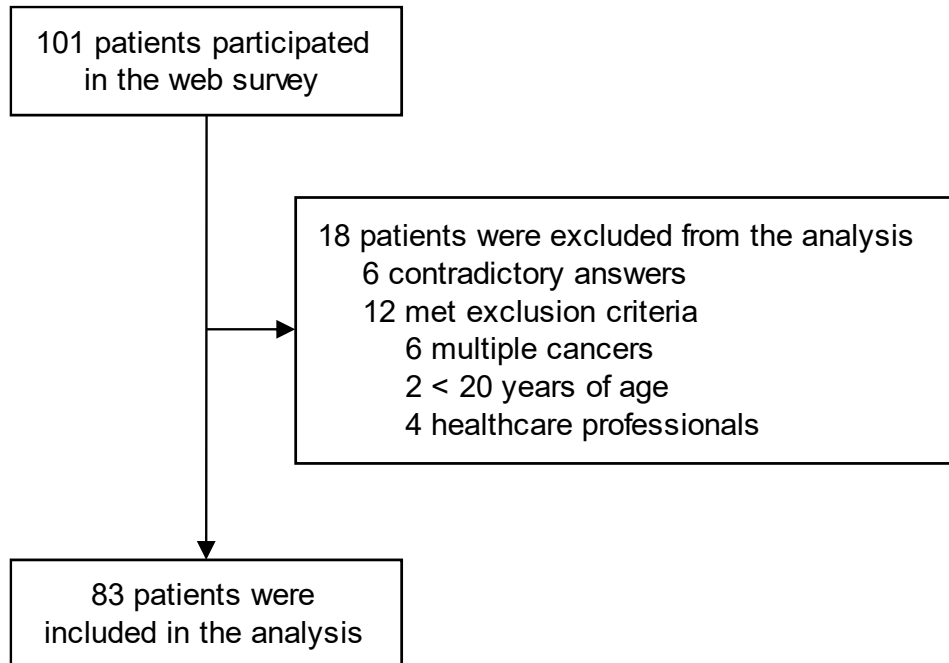

**Figure S1. Flowchart showing recruitment of patients.**

Supplement: Supplementary file 1 [file cancers-16-01904-s001.zip › cancers-2960765-supplementary.pdf]
